# Major decline in marine and terrestrial animal consumption by brown bears (*Ursus arctos*)

**DOI:** 10.1038/srep09203

**Published:** 2015-03-17

**Authors:** Jun Matsubayashi, Junko O. Morimoto, Ichiro Tayasu, Tsutomu Mano, Miyuki Nakajima, Osamu Takahashi, Kyoko Kobayashi, Futoshi Nakamura

**Affiliations:** 1 Center for Ecological Research, Kyoto University, 2-509-3 Hirano, 520-2113 Otsu, Shiga, Japan; 2 Laboratory of Forest Ecosystem Management, Graduate School of Agriculture, Hokkaido University, Kita 9 jo, Nishi 9, Kitaku, 060-8589 Sapporo, Hokkaido, Japan; 3 Research Institute for Humanity and Nature, 457-4 Motoyama, Kamigamo, Kita-ku, 603-8047 Kyoto, Japan; 4 Environmental and Geological Research Department, Hokkaido Research Organization, Kita 19 jo, Nishi 12, Kitaku, 060-0819 Sapporo, Hokkaido, Japan; 5 Salmon and Freshwater Fisheries Research Institute, Hokkaido Research Organization, 3-373 Kita-Kashiwagi, 061-1433 Eniwa, Hokkaido, Japan; 6 Chitose Archaeological Operations Center, Chitose Board of Education, Chitose city, 42-1 Osatsu, 066-0001 Chitose, Hokkaido, Japan; 7 Laboratory of Wild Wildlife Management, United Graduate School of Agricultural Science, Tokyo University of Agriculture and Technology, 3-8-1 Harumi, 183-8538 Fuchu, Tokyo, Japan

## Abstract

Human activities have had the strongest impacts on natural ecosystems since the last glacial period, including the alteration of interspecific relationships such as food webs. In this paper, we present a historical record of major alterations of trophic structure by revealing millennium-scale dietary shifts of brown bears (*Ursus arctos*) on the Hokkaido islands, Japan, using carbon, nitrogen, and sulfur stable isotope analysis. Dietary analysis of brown bears revealed that salmon consumption by bears in the eastern region of Hokkaido significantly decreased from 19% to 8%. In addition, consumption of terrestrial animals decreased from 56% to 5% in western region, and 64% to 8% in eastern region. These dietary shifts are likely to have occurred in the last approximately 100–200 years, which coincides with the beginning of modernisation in this region. Our results suggest that human activities have caused an alteration in the trophic structure of brown bears in the Hokkaido islands. This alteration includes a major decline in the marine-terrestrial linkage in eastern region, and a loss of indirect-interactions between bears and wolves, because the interactions potentially enhanced deer predation by brown bears.

Human intervention in natural ecosystems has been highly influential since the last glacial period^1^. Human development has resulted in the mass extinction of species^2^, deterioration of ecosystems^3^ and global climate change^4^. Among many types of human-caused ecosystem impacts, the alteration of food web structures is one of the most difficult alterations to assess^5^ because information regarding the food web composition during prehuman conditions is scarcely available. However, stable isotope techniques can overcome these problems by reconstructing the historical feeding habits of omnivores and generalist predators with large home ranges and acting as a good indicator of food web structures^5^.

Brown bears (*Ursus arctos*), which are widely distributed throughout the northern hemisphere, are recognised as opportunistic omnivores that flexibly change their feeding habits depending on the availability of dietary resources^6^. Therefore, when the availability of certain resources in terrestrial ecosystems changes, the contribution of those resources to the brown bears' diet should change accordingly. In other words, the historical dietary information of brown bears should record changes in the food web structures of local terrestrial ecosystems over a given time period. Here, we investigated the millennium-scale diet changes in brown bears in the Hokkaido islands, Japan, using carbon, nitrogen and sulfur stable isotope analysis, and we assessed the impacts of human development on the food web structures of these terrestrial ecosystems.

The brown bear habitat extends throughout the Hokkaido islands. Significant human development began in this area in approximately mid-19th century and has proceeded rapidly in the last 200 years. Recent dietary studies of Hokkaido brown bears have suggested potential alterations in their feeding habits. First, brown bears generally eat large amounts of salmon if it is available^7^,^8^. However, although both brown bears and salmon are found in Hokkaido, salmon consumption by brown bears in this area is considered to be minimal^9^. To clarify the reason for the low rate of salmon consumption, Matsubayashi et al.^10^ examined spatial differences of salmon consumption by brown bears using stable isotope analysis in bone collagen and suggested that land development in Hokkaido has restricted salmon-bear interactions. Second, the abundance of Sika deer (*Cervus nippon*) has changed in Hokkaido over the last decades, and several dietary studies based on stomach content analysis showed that such changes in deer availability are reflected in the brown bear diet^11^,^12^. Thus, the Hokkaido islands should be a suitable region to assess the relationship between human impacts and bear feeding habits.

In the Hokkaido islands, many animal bones, including brown bear bones, have been excavated near the remains of the indigenous Ainu people (Fig. 1). Stable isotope ratios of the collagen in animal bones reflect their feeding habits over several years^13^. We sampled bone collagen from the western and eastern regions in Hokkaido (Fig. 1, Supplementary Fig. 1), which have different levels of salmon and deer abundance. In the western area, only chum salmon (*Oncorhynchus keta*) run upstream from October to February, and the abundance of Sika deer is relatively low^14^. In contrast, in the eastern area, both pink salmon (*O. gorbuscha*) and chum salmon run upstream from August to October and from October to February, respectively, and the deer abundance is relatively high^14^. Brown bear bones from each region were divided into three time bins based on their time of death. These phases were defined based on the developmental chronology of the Hokkaido islands and are labelled as Period 1 (pre-development phase; before 1890 for Western area and 1920 for Eastern area), Period 2 (early phase of development; 1931–1942) and Period 3 (post-development phase; after 1996). In addition, we measured the stable isotope values of potential diet items of Hokkaido brown bears to assist in the interpretation of the isotopic values in brown bears.

## Results

We measured the stable carbon (δ^13^C), nitrogen (δ^15^N) and sulfur (δ^34^S) isotope ratios in bear collagen and potential diet items (C_3_ herbs, C_3_ fruits, corn, terrestrial animals and salmon^10^) of brown bears (Fig. 2, Supplementary Table 1, 2). The δ^13^C, δ^15^N and δ^34^S values of the potential diet items were compared using the K-nearest-neighbour randomisation test^15^ (Bonferroni-adjusted *P* < 0.003) in each area. In the western area, no significant differences were detected between C_3_ herbs and C_3_ fruits; therefore, we combined these groups as C_3_ plants. In the eastern area, all comparisons between diet groups showed substantial differences in the isotopic values.

For the western region, significant differences among Period 1–3 bears were found for the δ^13^C (Kruskal-Wallis test; W = 52.852, 2 degrees of freedom (df), *P* < 0.050), δ^15^N (W = 41.403, 2 df, *P* < 0.050) and δ^34^S (W = 21.691, 2 df, *P* < 0.050) results. The bears in Period 1 showed higher δ^13^C levels than in Periods 1 and 2, and the Period 2 bears showed higher δ^13^C levels than the Period 3 bears (Steel-Dwass multiple comparisons test; *P* < 0.050, Supplementary Tables 1 and 3). The bears in Period 1 showed higher δ^15^N levels than in Periods 1 and 2. The Periods 1 and 2 bears showed higher δ^34^S levels than in Period 3. For the eastern region, there were significant differences among Period 1–3 bears in their δ^13^C (W = 54.787, 2 df, *P* < 0.050), and δ^15^N (W = 45.253, 2 df, *P* < 0.050) levels but not in their δ^34^S levels (W = 3.687, 2 df, *P* = 0.158). The bears in Period 1 showed higher δ^13^C levels than in Periods 1 and 2 (Supplementary Table 1 and 3). The bears in Period 1 showed higher δ^15^N levels than in Periods 1 and 2.

We estimated the contribution of each diet item to the bears' diet in each time bin using a Bayesian mixing model, Stable Isotope Analysis in R (SIAR^16^). The carbon and nitrogen isotope values of diet items change temporally; therefore, corrected δ^13^C and δ^15^N values were used for the SIAR (Supplementary Table 4 and Supplementary Text 1). In contrast, δ^34^S values of diet items were used for SIAR without temporal correction because there is no positive evidence of temporal variation in δ^34^S. In the western area, bears from Periods 2 and 3 mainly depended on C_3_ plants (mode: 84% for Period 2 and 85% for Period 3), and the consumption of terrestrial animals and salmon was low (7% and 1% for Period 2 and 5% and 0% for Period 3, respectively) (Supplementary Table 5 and Fig. 3). By contrast, bears in Period 1 consumed a substantially lower proportion of C_3_ plants (41%) relative to those in Periods 2 and 3, and the most dominant diet item was terrestrial animals (56%); the consumption of salmon (3%) was low, similar to that of the other periods. In the eastern area, bears in Periods 2 and 3 showed a high dependence on C_3_ herbs (35% for Period 2 and 54% for Period 3) and C_3_ fruits (30% for Period 2 and 17% for Period 3). Terrestrial animals represented relatively higher proportion in Period 2 (27%) but a low proportion in Period 3 (8%). The proportions of salmon (5% and 8%) and corn (5% and 9%) were relatively low for Periods 2 and 3, respectively (Supplementary Table 5 and Fig. 3). By contrast, Period 1 bears mainly depended on terrestrial animals (64%) and salmon (19%), and the contribution of plant matter was relatively low (12% for C_3_ herbs and 1% for C_3_ fruits).

Our limited sample size of Period 1 bears made it difficult to compare isotopic values for each archaeological site (Supplementary Table 6). Therefore, we made time-series plots for each stable isotope (Fig. 4 and Supplementary Fig. 3) and read out the change-points of bear isotopic values. The temporal changes in the three stable isotope values suggested that the decline of the trophic level of brown bears began in 1800–1900 in both the western and eastern areas (Fig. 4 and Supplementary Fig. 3).

## Discussion

The δ^13^C and δ^15^N values of the bear bone collagen showed significant temporal shifts between Periods 1 and 2. The temporal changes in the δ^13^C values can be explained by the Suess effect because the difference between Periods 1 and 2 (0.9‰ for the western area and 1.5‰ for the eastern area) and between Periods 1 and 3 (1.7‰ for the western area and 2.1‰ for the eastern area) are consistent with the expected Suess effect (1.3‰ and 1.6‰, respectively, see Supplementary Text 1). In contrast, the temporal shifts in the δ^15^N values of bears between Periods 1 and 2 (2.1‰ for the western area 2.6‰ for the eastern area), and between Periods 1 and 3 (2.9‰ for the western area and 3.7‰ for the eastern area) cannot be accounted for by temporal differences in δ^15^N values of their diet (at a maximum 0.4‰, see Supplementary Text 1). Therefore, the temporal decline of δ^15^N values of bears reflected differences in the brown bear's feeding habits, especially for the consumption of animal tissue. Temporal shifts in the δ^34^S values were only found in the western area, which can also be attributed to differences in feeding habits between Periods.

Our stable isotope analysis illustrated that a major decline in animal consumption by brown bears occurred from Period 1 to Period 3 in the eastern and western area of Hokkaido (Fig. 4). Why did these declines in animal consumption occur? We considered whether the spatial biases of the sampled bears affected animal consumption. Differences in habitat quality (e.g., coastal areas or inland areas) may influence the availability of animal matter (especially salmon) for brown bears. Because the capture locations of the bears from Periods 1 and 2 were uncertain, discussing the spatial bias of our historical samples is difficult. However, in the case of Period 1 bears, we can assume that bears were captured near the archaeological sites where they were found because it was difficult for pre-modern people to move away from their home village. Almost half of our data for Period 1 bears came from inland sites (Bifue-Iwakage for the western and Nijibetsu-Suwan for eastern areas; see Supplementary Fig. 1). Therefore, our Period 1 bears were not spatially constrained. Most Period 3 bears in the eastern area were sampled from the coastal area of the Shiretoko peninsula, which is located on the northeastern tip of Hokkaido. Because the Shiretoko peninsula is an ideal region in Hokkaido for brown bears to catch salmon, the salmon consumption of Period 3 bears in the eastern area is, if anything, likely to be an overestimation. Thus, the observed dietary shift in Hokkaido brown bears was not caused by the spatial biases of the sampling locations.

The Ainus had a custom of raising captured bear cubs in captivity by feeding them high-protein foods until they arrive at the age of two or three years^17^,^18^. This practice could be a possible reason for the higher animal protein consumption by Period 1 bears because some of the Period 1 bears were not adults (3–4 yrs) nor were age data available (Supplementary Table 7). If these bears were affected by the Ainu's feeding activity, they should show higher δ^15^N values than adult bears. However, in our results, there were no significant differences in δ^15^N values between adults and other bears for either region (Wilcoxon test, *P* > 0.050). For this reason, we concluded that the Ainu's feeding activity did not cause the observed higher animal protein consumption of Period 1 bears.

Our data include bears killed for nuisance control and sport, and these differences in the sampling methodology may have influenced our results. In general, dietary analyses based only on recent dietary information, such as stomach contents at the time of death, tend to overestimate the proportion of the anthropogenic diet^19^. However, the stable isotope analysis of bone collagen reflects dietary records several years before death^13^. In addition, bears are commonly controlled by the Hokkaido government, and it is difficult for bears to continue to consume anthropogenic prey such as garbage and agricultural crops for an extended period. Thus, the stable isotope ratios in the bone collagen of Hokkaido brown bears reflect their dietary habits prior to the nuisance activities, and biases in feeding habits that can be attributed to the sampling method should not influence the results.

Determining the time when the major dietary shift occurred is important to understanding why bears stopped consuming animal protein. Temporal changes in the δ^15^N values suggested that the decline in the trophic level of brown bears started in approximately 1800–1900 in both the western and eastern areas (Fig. 4). This period correlates with the beginning of the Meiji period (1868) when Hokkaido islands had started to be developed intensively. Development actions that could impact salmon consumptions by bears include changes in land use, river improvements and large-scale industrial fishing. Brown bears generally tend to avoid human facilities such as paved roads^20^. Changes in land use such as the construction of paved roads, expansion of urban areas and farmland are primary factors for reclamations and occur concurrently with the progression of development. In addition, the development in Hokkaido initially occurred along the coast. Thus, land-use changes in coastal areas makes catching salmon at downstream sites difficult for bears. In addition, the use of trap nets by the salmon fishing industry began in the mid-19th century in Hokkaido^21^. Salmon fishing before the introduction of trap nets was mainly performed in river valleys^22^. These large-scale fishing practices had strong impacts on salmon populations and resulted in dramatically decreased salmon catches after 1890^21^. On the Ishikari River, where the statistical data for salmon catches after 1868 is available, the annual mean salmon catch from 1868 to 1889 (920,540 salmon per year) was reduced to less than one third of that value from 1890 to 1903 (295,669) as a result of over fishing^23^. Similar low abundance of salmon was observed throughout Hokkaido during 1870 to 1970 (annual mean salmon catch < 5,000,000 per year)^24^. Then, the salmon catch increased rapidly after 1970 (over 40,000,000 per year after 1990)^24^, as observed in other Pacific regions^25^. This increase in salmon catch can be attributed to the expansion of salmon hatcheries^26^ and the climatic regime shift in the Pacific Ocean^27^ in approximately 1970. Although the salmon abundance in the Hokkaido islands at present is relatively high, almost all salmon are caught by trap nets in the coast or downstream, and only the few salmon that can escape or avoid these traps run up their natal stream. Thus, the expansion of the large-scale fishing industry would have decreased the availability of salmon to brown bears. For these reasons, our results strongly suggest that human impacts after 19th century resulted in a major decline in salmon consumptions by Hokkaido brown bears.

In addition to salmon, the contribution of terrestrial animals to the bears' diet also significantly decreased from Period 1 to Period 3 in both areas. Sika deer are terrestrial animals commonly consumed by brown bears^9^. There are two factors that potentially decreased the consumption of deer by brown bears during 1800–1900. First, a mass death of Sika deer occurred at the end of the 19th century as a result of overhunting and two heavy snows^28^. Thus, deer abundance in Period 2 was strictly limited, and brown bears could not consume them very often. Since then, populations of Sika deer have rapidly recovered^14^, and therefore the abundance of deer cannot explain the observed low contribution of terrestrial animals to bear diet in Period 3. The second factor, which may account for the low deer consumption in Period 3, is the extinction of the Hokkaido wolf (*Canis lupus hattai*)^29^,^30^. Brown bears in Hokkaido are rarely capable of hunting adult deer by themselves; however, studies in Yellowstone ecosystems have shown that bears can usurp wolf-hunted ungulates when bears and wolves inhabit the same location because wolves cannot compete against larger brown bears^31^,^32^. Therefore, the presence of wolves could have increased the consumption of deer by brown bears. Wolves in Hokkaido became extinct due to the overhunting and the mass death of Sika deer at the end of the 19th century, and this time period corresponds to the drastic isotope shift observed in Hokkaido brown bears after 1800. Our results suggested that the indirect effect of the loss of wolves prevented bears from consuming deer. At present in the Hokkaido islands, increasing deer populations cause many problems both in ecosystems, agriculture and forestry^33^. To date, the present expansion of deer populations has been explained by extinction of wolves^34^, however, we have shown that brown bears also previously contributed to the repression of deer populations. We suggest that the important factors to controlling Sika deer populations in Hokkaido are not only predation by wolves but also the indirect effects between wolves and brown bears.

In this study, the historical decline in marine and terrestrial animal consumption by Hokkaido brown bears was demonstrated, and a relationship between the observed dietary shifts and human impacts was strongly suggested. These findings also imply that brown bears can be an ecological indicator which reflects the alteration of their food webs. Although the loss of a major source of dietary nutrients could be a factor in the extinction of large omnivores^19^, animal protein such as salmon and deer should not be an essential resource for brown bears, because brown bears in Hokkaido have sustained their populations for the last 200 years despite their decreasing animal consumption. However, the observed dietary shifts in Hokkaido brown bears may cause several ecological problems, such as a shift in nutrient cycling and human-bear conflicts. Salmon consumption by brown bears creates an important linkage between the marine and terrestrial networks^35^,^36^. Therefore, the decline of marine derived nutrients (MDN) via salmon predation by brown bears may change the nutritional conditions of riparian vegetation and small animals that are linked in the food chain^35^,^37^. Although similar limitations in MDN transfer by human activity have been reported in a lake ecosystem in Alaska^38^, no other studies have shown a human-induced decline in MDN. Most studies have focused on the importance of MDN to terrestrial or freshwater ecosystems^39^,^40^; however, the anthropogenic impact on MDN transfer should receive more attention in subsequent studies. In addition, Hokkaido brown bears face food shortages and consume crops more frequently in late summer^41^, which is the spawning season for salmon that run upstream. Thus, the restriction of salmon likely increase human-bear conflicts. We have shown major alterations in the trophic structures of Hokkaido brown bears, and similar human-induced changes in feeding habits have also been reported for polar bears^42^, owls in Europe^43^, Atlantic cod in the southern Gulf of St Lawrence^44^, and the Hawaiian petrel in the Pacific Ocean^5^. These studies suggest that such invisible changes in the trophic structures of top predators may have been occurring worldwide since the beginnings of human settlement.

## Methods

### Study site

**Figure 1:**
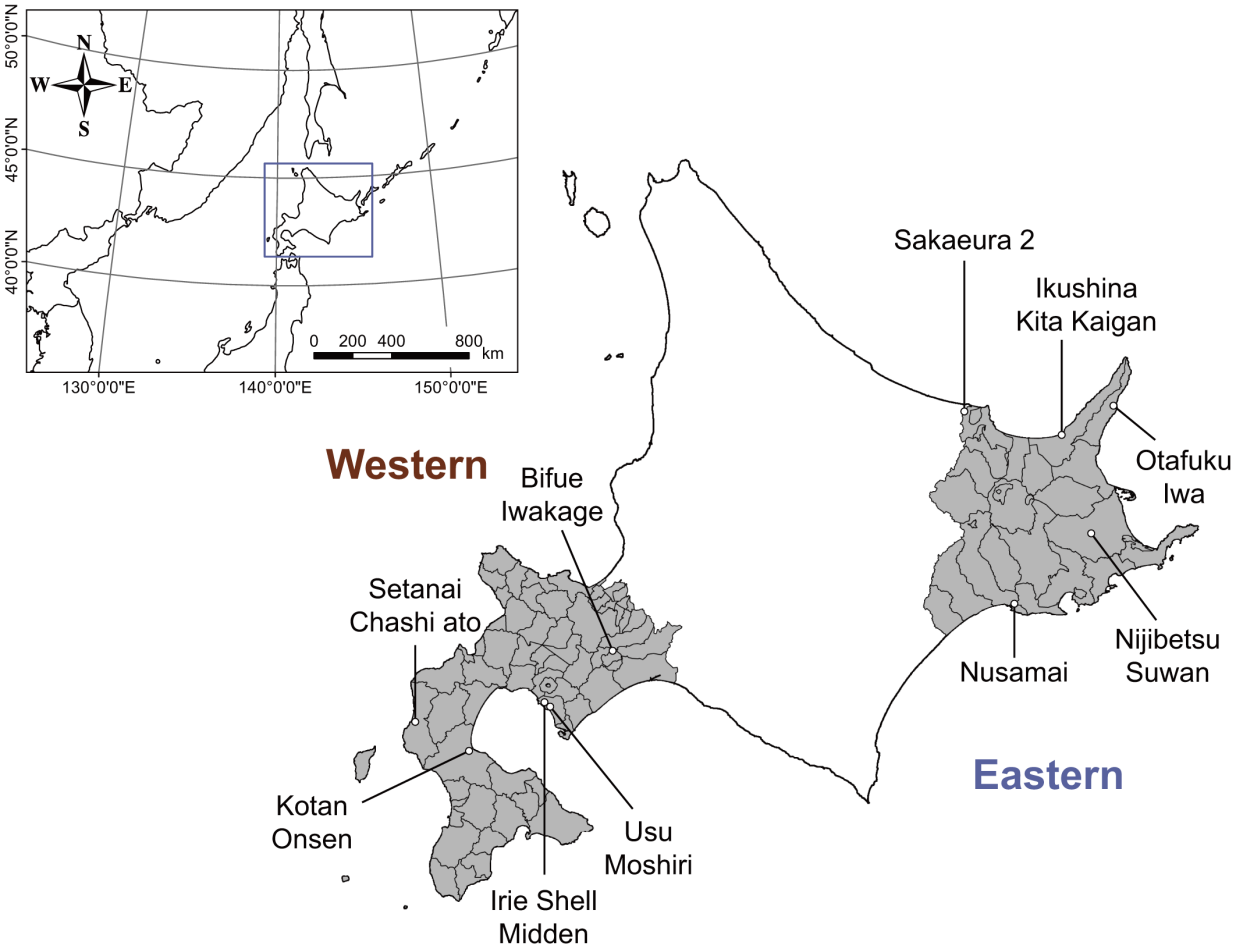
The locations of the Hokkaido islands and each archaeological sites. This figure was made using GIS software (ArcGIS Desktop 10.2.1).

**Figure 2:**
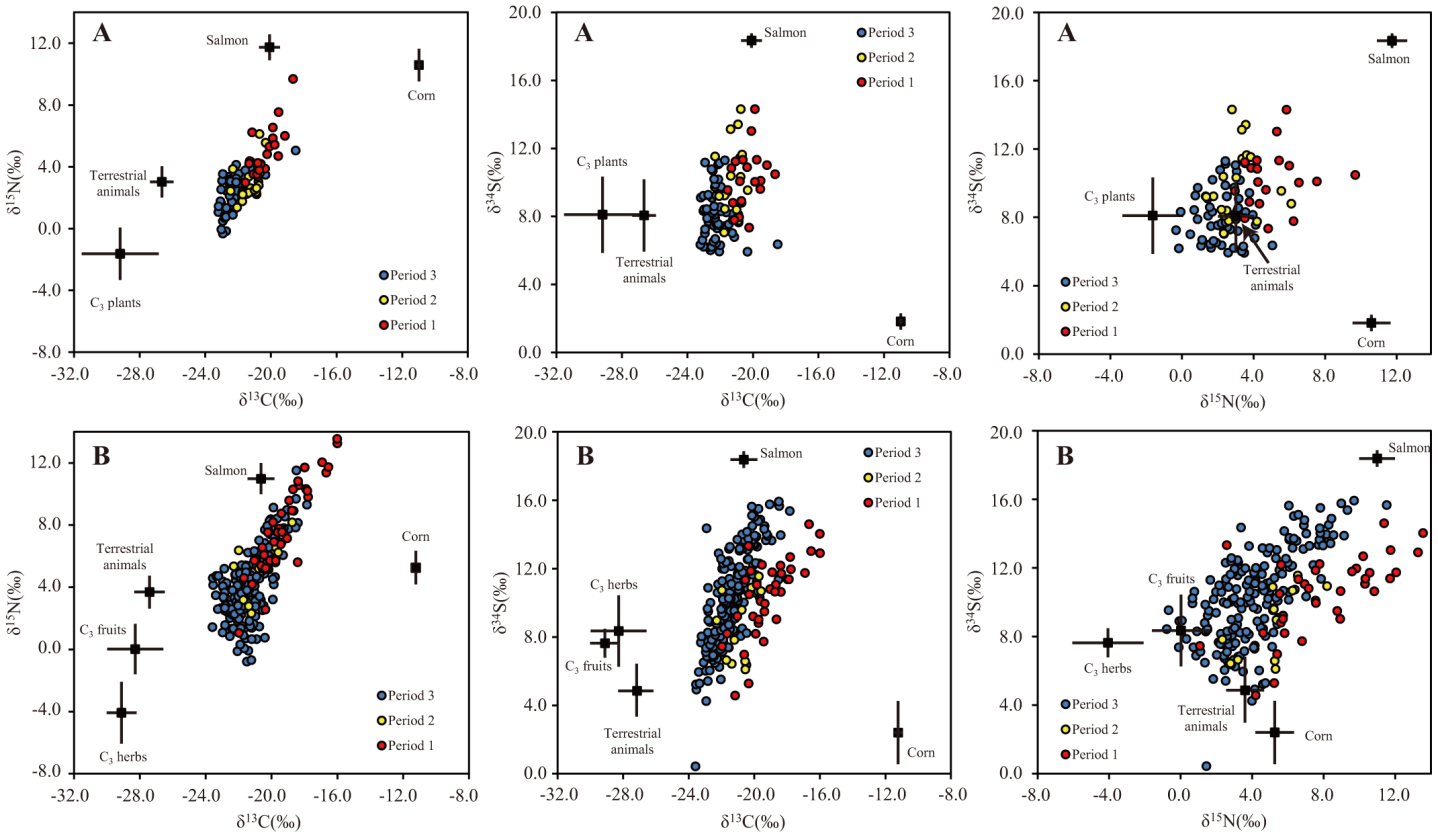
C, N and S stable isotope ratios in bone collagen and potential prey items (mean ± SD) in Hokkaido brown bears. (A) represents the isotopic values in the western area, and (B) represents the eastern area.

**Figure 3:**
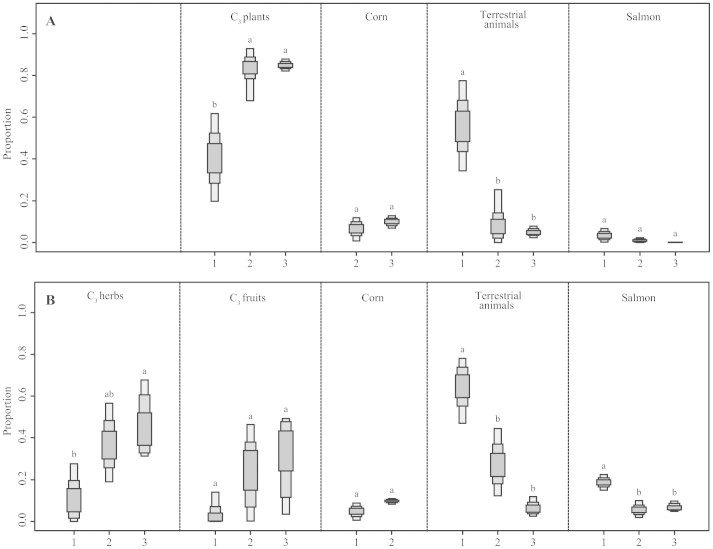
Historical variation of diet components of Hokkaido brown bears as illustrated by SIAR distributions. Box plots illustrate high and low 95%, 75% and 50% high density ranges (hdr) of proportions of each prey item. Numbers under the horizontal axis refer to each time bin. Period 1 represents the era before 1890 for Western area and 1920 for Eastern area, Period 2 represents 1931–1942, and Period 3 represents the period after 1996. Different letters indicate significance based on the overlap of 95% hdrs (see Supplementary Table 5). (A) represents the dietary contributions of bears in the western area, and (B) represents the eastern area.

**Figure 4:**
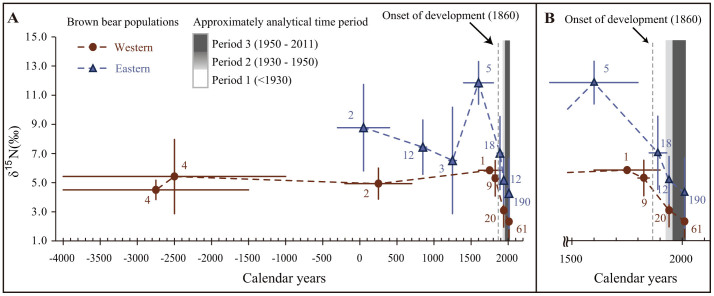
δ^15^N values of historical bone collagen for two Hokkaido brown bear populations. The average age and isotopic composition of each time bin ± SD for δ^15^N values is shown; the age range of each bear group was based on the age of the site at which their bones were found, and the sample size was noted. The age of bear groups earlier than 1920 was determined by the archaeological chronology of each archaeological site. (A) represents a δ^15^N shift within the whole period, and (B) is an expanded plot after 1500. Grey shading indicates time bins. Stippled lines connecting data points are for visualisation purposes; isotopic shifts between time bins may have occurred nonlinearly.

This study was conducted in the western and eastern areas of Hokkaido Island, the northernmost island of Japan (Fig. 1 and Supplementary Fig. 1). The mean annual temperature in the western area is 8.9°C (a representative value from Sapporo city), and the mean annual precipitation is 1106.5 mm. Because the western area is adjacent to the main islands of Japan, and a part of the area already has been developed in 1890, which was the earliest development in Hokkaido. The mean annual temperature in the eastern area is 6.5°C, and the mean annual precipitation is 787.6 mm (a representative value from Abashiri city). The eastern area includes the Shiretoko region, which was placed on the World Heritage List in July 2005 as a Natural Heritage Site. It is valued for its unique ecosystems that are shaped by interactions between the marine and terrestrial ecosystems^45^. Development in the eastern area began to progress during 1868–1920, which was the slowest rate of progression in Hokkaido. Primary reclamation began after the 20th century, and several regions, such as the distal area of the Shiretoko peninsula, are still intact. Genetic studies analyzing bear mtDNA have shown that brown bears in Hokkaido divided into three large populations from the western, central and eastern regions from more than 300,000 years ago^46^. The area classifications in this study mostly correspond with the genetically distinct western and eastern populations. Although the western and eastern populations of this study are genetically different, some males with a relatively large home range^47^,^48^ may be able to move across the populations.

### Sample collection

Bone fragments of brown bears were sampled from local museums (Supplementary Table 6). The archaeological age of these bears was consistent with the age of the site where their bones were found. Age of each archaeological site is determined based on the archaeological find and geological layers of the site. Bones of modern bears were obtained from the Hokkaido Institute of Environmental Science (HIES), which has collected the thigh bones of bears killed in nuisance control programs and for sport. The experiments and the collection of modern bear bone samples were approved by the Hokkaido Research Organization, Local Independent Administrative Agency. We excluded bears less than or equal than 2 years old because the mother's milk would influence the nitrogen isotope signature^49^. We were not able to assess the age data of several bears due to the lack of teeth. In this case, we only used samples that were clearly adult bears based on their bone size. The potential diet items of Hokkaido brown bears were sampled from multiple points in the eastern and western areas. Then, prey items were provisionally categorised as C_3_ herbs, C_3_ fruits, C_4_ plants, terrestrial animals and salmon based on a previous study^10^. Corn was used as an indicator of the consumption of agricultural crops because corn accounted for over half of the crops eaten by Hokkaido brown bears in each region^11^.

### Sample preparation and stable isotope analysis

The deer and salmon tissues were dried at 60°C for at least 2 days and then ground in a mortar. To extract the lipids, the dried, powdered samples were placed in glass centrifuge tubes and immersed in a mixed solvent of methanol:chloroform (1:1). The samples were then mixed for 30 s, left undisturbed for at least 1 hour and centrifuged for 10 min at 2500 rpm. We repeated this process 3 times. The plant tissues and ants were also dried at 60°C for at least 2 days, but the lipids were not extracted because these organisms generally contain extremely low lipid levels. Instead, these organic tissues were ground in a mortar and then placed in tin capsules for elemental and stable isotope analyses. The bear thigh bones were ground using a drill, blender or micro-grinder cooled with liquid nitrogen. The bone collagen was then extracted according to the methods of Schoeninger and DeNiro^50^.

The carbon, nitrogen and sulfur isotope ratios were expressed in δ notation based on the international standard scale, according to the following equation (1):
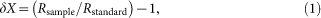
where X is ^13^C, ^15^N or ^34^S; *R*_sample_ corresponds to the ^13^C/^12^C, ^15^N/^14^N or ^34^S/^32^S ratio of the measured samples; and *R*_standard_ for ^13^C/^12^C was Vienna Pee Dee Belemnite (VPDB), that for ^15^N/^14^N was atmospheric nitrogen (AIR), and that for ^32^S/^34^S was Vienna Canyon Diablo Troilite (VCDT). The carbon and nitrogen stable isotope ratios were measured with commercially used equipment (ANCA-IRMS, Europa Scientific Integra, UK) at the University of California, Davis. The sulfur stable isotope ratios were measured using a Delta V Plus mass spectrometer (Thermo Fisher Scientific, Waltham, Massachusetts, USA) connected to a Flash EA 2000 elemental analyser. The overall measurement error was estimated to be less than 0.1‰ for δ^13^C, 0.3‰ for δ^15^N and 0.5‰ for δ^34^S. In addition, carbon and nitrogen stable isotope data for diet items and modern bears of the eastern region were obtained from Matsubayashi et al^10^. Stable isotope values of past bone collagen are sometimes affected by bone diagenesis^51^. To exclude the collagen that was influenced by diagenesis, we applied several indicators of pure collagen: an atomic C:N ratio from 2.9 to 3.6^52^, an atomic N:S ratio from 300 to 900, an atomic N:S ratio from 100 to 300, and a weight %S between 0.15 and 0.35^53^. Then, samples outside the acceptable limits were excluded from further analyses (Supplementary Table 7).

### Definition of the three time bins for historical bear samples

We used three time bins based on the developmental progress of Hokkaido. Before the mid-19th century, the indigenous Ainu people lived in Hokkaido and mostly depended on hunting and gathering. After 1868, people from mainland Japan moved to the Hokkaido islands and started the process of reclamation. Development in the large part of Western and Eastern area were started in 1890 and 1920, respectively^54^. Therefore, the era before 1890 for Western area and 1920 for Eastern area were defined as Period 1 and was minimally influenced by human activities. We categorised the era from 1931 to 1942 as Period 2 to reflect the dietary information of bears in the early phase of development. The development of Hokkaido was clearly advanced prior to 1996. Therefore, we defined the period after 1996 as Period 3, the post-development phase.

### Mixing model analysis and statistical analysis

We employed a K-nearest-neighbour randomisation test^15^ (Bonferroni-adjusted *P* < 0.005) to investigate whether the stable isotope ratios of the various food types differed significantly from each other. Groups that did not show significant differences were combined (Supplementary Table 3). In addition, corn was excluded from the potential diet items for Period 1 because it was imported to Hokkaido at the end of the 19th century. Historical differences in the stable isotope values for bear bones were tested using the Kruskal–Wallis test followed by the Steel–Dwass multiple comparisons test (*P* < 0.050) among the three time periods. We evaluated the proportional contribution of each food resource to the bear groups using a Bayesian isotopic mixing model available as an open-source R package, Stable Isotope Analysis in R (SIAR)^16^. For the dietary estimation by SIAR, δ^13^C and δ^15^N values of prey items were corrected for the temporal isotopic shift (see Supplementary Text 1) because there should be temporal isotopic shifts for the diet items in Periods 1 and 2 compared to isotopic values in Period 3. By contrast, δ^34^S was used for SIAR without any temporal correction because there is no positive evidence of temporal variation in δ^34^S. The SIAR model was fitted through a Markov Chain Monte Carlo (MCMC) procedure to simulate plausible values for the dietary proportion of each source consistent with the data and based on a Dirichlet prior distribution^14^. The SIAR MCMC was run for 1,000,000 iterations. The first 100,000 samples were discarded to avoid the possible effects of the starting value. We assigned an elemental concentration (%C, %N and %S)^55^ because the C, N and S concentrations in the prey items of brown bears varied (Supplementary Table 4). Because different tissues incorporate isotopes at different rates, we applied a correction factor for bone collagen to incorporate the isotopic discrimination between consumer and prey before generating the model. We used a fractionation of 5.0‰ ± 1.5‰ SD^50^,^51^,^56^,^57^,^58^ for δ^13^C, 3.0‰ ± 1.5‰ SD for δ^15^N^50^,^59^,^60^,^61^,^62^, and 1.0‰ ± 0.5‰ SD for δ^34^S^63^,^64^,^65^. All statistical analyses were conducted using R (R Core Development Team, R Foundation for Statistical Computing, Vienna).

## Author Contributions

J.M., J.O.M., I.T. and F.N. conceived the study and secured funding. M.N. and K.K. partially collected diet items of brown bears. T.M. and O.T. partially collected modern and ancient brown bear samples, respectively. I.T. set an isotope ratio mass spectrometer to measure sulfur stable isotope ratios. J.M. led the analysis of data and authorship of the manuscript. All authors contributed to the writing of this manuscript.

## Supplementary Material

Supplementary Information Supplementary Information

## References

[b1] Estes J. A. *et al.* Trophic downgrading of planet earth. Science 333, 301–306 (2011). 21764740 10.1126/science.1205106

[b2] Tilman D., May R. M., Lehman C. L. & Nowak M. A. Habitat destruction and the extinction debt. Nature 371, 65–66 (1994).

[b3] Jackson J. B. C. *et al.* Historical overfishing and the recent collapse of coastal ecosystems. Science 293, 629–638 (2001). 11474098 10.1126/science.1059199

[b4] Intergovernmental Panel on Climate Change. Climate Change 2007: The Physical Science Basis. Contribution of Working Group I to the Fourth Assessment Report of the Intergovernmental Panel on Climate Change (Cambridge University Press, Cambridge, U.K., 2007).

[b5] Willey A. E. *et al.* Millennial-scale isotope records from a wide-ranging predator show evidence of recent human impact to oceanic food webs. Proc. Natl. Acad. Sci. USA 110, 8972–8977 (2013). 23671094 10.1073/pnas.1300213110 PMC3670381

[b6] Mowat G. & Heard D. C. Major components of grizzly bear diet across North America. Can. J. Zool. 84, 473–489 (2006).

[b7] Hilderbrand G. V., Thomas A. H., Charles T. R. & Charles C. S. Role of brown bears (*Ursus arctos*) in the flow of marine nitrogen into a terrestrial ecosystem. Oecologia 121, 546–550 (1999). 10.1007/s004420050961 28308364

[b8] Reimchen T. E. Some ecological and evolutionary aspects of bear–salmon interactions in coastal British Columbia. Can. J. Zool. 78, 448–457 (2000).

[b9] Sato Y., Mano T. & Takatsuki S. Stomach contents of brown bears *Ursus arctos* in Hokkaido, Japan. Wildlife Biol. 11, 133–144 (2005).

[b10] Matsubayashi J., Morimoto J., Mano T., Achyut A. & Nakamura F. Using stable isotopes to understand the feeding ecology of the Hokkaido brown bear (*Ursus arctos*) in Japan. Ursus 25, 87–97 (2014).

[b11] Sato Y., Aoi T., Kaji K. & Takatsuki S. Temporal changes in the population density and diet of brown bears in eastern Hokkaido, Japan. Mamm. study 29, 47–53 (2004).

[b12] Kobayashi K., Sato Y. & Kaji K. Increased brown bear predation on sika deer fawns following a deer population irruption in eastern Hokkaido, Japan. Ecol. Res. 27, 849–855 (2012).

[b13] Stenhouse M. J. & Baxter M. S. in Radiocarbon Dating (eds Berger, R. & Suess, H. E.) 324–341 (University of California Press, Los Angeles, U.S.A., 1976).

[b14] Yamamura K. *et al.* Harvest-based Bayesian estimation of sika deer populations using state-space models. Popul. Ecol. 50, 131–144 (2008).

[b15] Rosing M. N., Ben-David M. & Barry R. P. Analysis of stable isotope data: a K nearest-neighbors randomization test. J. Wildlife Manage. 62, 380–388 (1998).

[b16] Parnell A., Inger R., Beahop S. & Jackson A. L. Source partitioning using stable isotopes: coping with too much variation. PLoS ONE 5, e9672 (2010). 20300637 10.1371/journal.pone.0009672 PMC2837382

[b17] Chaix L., Bridault A. & Picavet R. A tamed brown bear (*Ursus arctos L.*) of the Late Mesolithic from La Grande-Rivoire (Isère, France)? Journal of Archaeological Science 24, 1067–1074 (1997).

[b18] Hallowell A. I. Bear ceremonialism in the northern hemisphere. American Anthropologist 28, 120–131 (1926).

[b19] Hilderbrand G. V. *et al.* Use of stable isotopes to determine diets of living and extinct bears. Can. J. Zool. 74, 2080–2088 (1996).

[b20] Olson T. L., Gilbert B. K. & Squibb R. C. The effects of increasing human activity on brown bear use of an Alaskan river. Biol. Conserv. 82, 95–99 (1997).

[b21] Nagata M. *et al.* An overview of salmon enhancement and the need to manage and monitor natural spawning in Hokkaido, Japan. Environ. Biol. Fish. 94, 359–361 (2011).

[b22] Okada A. Maritime adaptations in Hokkaido. Arctic Anthropol. 35, 340–349 (1998).

[b23] Ishikari City. History of Ishikari: Middle Volume 1. 226–229 (Suda make-up company, Sapporo, japan, 1985) (in Japanese).

[b24] Kobayashi T. History of Salmon Stock Enhancement in Japan. (Hokkaido University Publication, Sapporo, Japan, 2009) (in Japanese).

[b25] Ruggerone G. T., Peterman R. M., Corner B. & Myers K. W. Magnitude and trends in abundance of hatchery and wild pink salmon, chum salmon, and sockeye salmon in the North Pacific Ocean. Mar. Coast. Fish. 2, 306–328 (2010).

[b26] Beamish R. J., Mahnken C. & Neville C. M. Hatchery and wild production of Pacific salmon in relation to large-scale, natural shifts in the productivity of the marine environment. J. Mar. Sci. 54, 1200–1215 (1997).

[b27] Morita K., Morita S. H. & Fukuwaka M. Population dynamics of Japanese pink salmon (*Oncorhynchus gorbuscha*): are recent increases explained by hatchery programs or climatic variations? Can. J. Fish. Aquat. Sci. 63, 55–62 (2006).

[b28] Nabata D., Masuda R., Takahashi O. & Nagata J. Bottleneck effects on the sika deer *Cervus nippon* population in Hokkaido, revealed by ancient DNA analysis. Zool. Sci. 21, 473–481 (2004). 15118235 10.2108/zsj.21.473

[b29] Kishida K. Notes on the Yesso wolf. Lansania 3, 72–75 (1931).

[b30] Inukai T. Eradication of Ezo wolf by humans. Zenshu Nihon Doubutsushi 5, 11–15 (1982) (in Japanese).

[b31] Alberta Environmental Protection. Wolves in Alberta: Their Characteristics, History, Prey Relationships and Management (Alberta Environmental Protection, Alberta, Canada, 1995).

[b32] Gunther K. A. & Smith D. W. Interactions between wolves and female grizzly bears with cubs in Yellowstone National Park. Ursus 15, 232–238 (2004).

[b33] Takatsuki S. Effects of sika deer on vegetation in Japan: A review. Biol. Conserv. 142, 1922–1929 (2009).

[b34] Nakajima N. Changes in distributions of wildlife in Japan. in Rebellion of Wildlife and Collapse of Forest (ed Forest and Environment Research Association, Japan) 57–68 (Shinrinbunka Association, Tokyo, Japan, 2007) (in Japanese).

[b35] Hilderbrand G. V., Thomas A. H., Charles T. R. & Charles C. S. Role of brown bears (*Ursus arctos*) in the flow of marine nitrogen into a terrestrial ecosystem. Oecologia 121, 546–550 (1999). 10.1007/s004420050961 28308364

[b36] Helfield J. M. & Naiman R. J. Keystone interactions: salmon and bear in riparian forests of Alaska. Ecosystems 9, 167–180 (2006).

[b37] Ben-David M., Hanley T. A. & Schell D. M. Fertilization of terrestrial vegetation by spawning Pacific salmon: the role of flooding and predator activity. Oikos 83, 47–55 (1998).

[b38] Schindler D. E. *et al.* Marine-derived nutrients, commercial fisheries, and production of salmon and lake algae in Alaska. Ecology 86, 3225–3231 (2005).

[b39] MacAvoy S. E., Macko S. A., Mclninch S. P. & Garman G. C. Marine nutrient contributions to freshwater apex predators. Oecologia 122, 588–573 (2000). 10.1007/s004420050980 28308350

[b40] Cederholm C. J., Kunze M. D., Murota T. & Sibatani A. Pacific Salmon Carcasses: Essential Contributions of Nutrients and Energy for Aquatic and Terrestrial Ecosystems. Fisheries 24, 6–15 (1999).

[b41] Sato Y. Feeding habits of brown bear: regional difference and annual variation. Mamm. Sci. 45, 79–84 (2005).

[b42] McKinney M. A., Peacock E. & Letcher R. J. Sea ice-associated diet change increases the levels of chlorinated and brominated contaminants in polar bears. Environ. Sci. Technol. 43, 4334–4339 (2009). 19603643 10.1021/es900471g

[b43] Bilney R. J., Cooke R. & White J. Change in the diet of sooty owls (*Tyto tenebricosa*) since European settlement: from terrestrial to arboreal prey and increased overlap with powerful owls. Wildlife Res. 33, 17–24 (2006).

[b44] Hanson J. M. & Chouinard G. A. Diet of Atlantic cod in the southern Gulf of St Lawrence as an index of ecosystem change, 1959–2000. J. Fish Biol. 60, 902–922 (2002).

[b45] Nakamura F. & Komiyama E. A challenge to dam improvement for the protection of both salmon and human livelihood in Shiretoko, Japan's third Natural Heritage Site. Landsc. Ecol. Eng. 6, 143–152 (2010).

[b46] Matsuhashi T., Masuda R., Mano T. & Yoshida M. C. Microevolution of the Mitochondrial DNA Control Region in the Japanese Brown Bear (*Ursus arctos*) Population. Mol. Biol. Evol. 16, 676–684 (1999). 10335661 10.1093/oxfordjournals.molbev.a026150

[b47] Yamanaka M., Okada H., Masuda T., Tsuruga H. & Kaji K. Study on habitat environment and habitat use of brown bears in Shiretoko Peninsula. in Landscape Ecological Studies on Basin Management Concerning about Conservation of High Nature Level Ecosystems (ed Hokkaido Forest Research Institute) 122–130 (Hokkaido Forest Research Institute, Sapporo, Japan, 1995) (in Japanese).

[b48] Mano T. Home range and habitat use of brown bears in the southwestern Oshima Peninsula, Hokkaido. Int. Conf. Bear. Res. Manage. 9, 319–325 (1994).

[b49] Bocherens H. *et al.* Diet reconstruction of ancient brown bears (*Ursus arctos*) from Mont Ventoux (France) using bone collagen stable isotope biogeochemistry (^13^C, ^15^N). Can. J. Zool. 82, 576–586 (2004).

[b50] Schoeninger M. J. & DeNiro M. J. Nitrogen and carbon isotopic composition or bone collagen from marine and terrestrial animals. Geochim. Cosmochim. Ac. 48, 625–639 (1984).

[b51] Hedges R. E. M. Bone diagenesis: An overview of processes. Archaeometry 44, 319–328 (2002).

[b52] DeNiro M. J. Postmortem preservation and alteration of in vivo bone collagen isotope ratios in relation to palaeodietary reconstruction. Nature 317, 806–809 (1985).

[b53] Nehlich O. & Richards M. P. Establishing collagen quality criteria for sulfur isotope analysis of archaeological bone collagen. Archaeol. Anthropol. Sci. 1, 59–75 (2009).

[b54] Historical Museum of Hokkaido. Progression of Hokkaido (Historical Museum of Hokkaido, Sapporo, Japan, 2000) (in Japanese).

[b55] Phillips D. L. & Koch P. L. Incorporating concentration dependence in stable isotope mixing models. Oecologia 130, 114–125 (2002). 10.1007/s004420100786 28547016

[b56] Vogel J. C. Isotopic assessment of the dietary habits of ungulates. S. Afr. J. Sci. 74, 298–301 (1978).

[b57] Von Schirnding Y., Vander Merwea N. J. & Vogel J. C. Influence of diet and age on carbon isotope ratios in ostrich eggshell. Archaeometry 24, 3–20 (1982).

[b58] Lee-Thorp J. A., Sealy J. C. & Merwe N. J. Stable carbon isotope ratio differences between bone collagen and bone apatite, and their relationship to diet. J. Archaeol. Sci. 16, 585–599 (1989).

[b59] DeNiro M. J. & Epstein S. Influence of diet on the distribution of nitrogen isotopes in animals. Geochim. Cosmochim. Ac. 45, 341–351 (1981).

[b60] Minagawa M. & Wada E. Stepwise enrichment of ^15^N along food chains: further evidence and the relation between δ^15^N and animal age. Geochim. Cosmochim. Ac. 48, 1135–1140 (1984).

[b61] Ambrose S. H. & DeNiro M. J. Reconstruction of African human diet using bone collagen carbon and nitrogen isotope ratios. Nature 319, 321–324 (1986).

[b62] Sealy J. C., van der Merwe N. J., Lee-Thorp J. A. & Lanham J. L. Nitrogen isotopic ecology in southern Africa: implications for environmental and dietary tracing. Geochim. Cosmochim. Ac. 51, 2707–2717 (1987).

[b63] McCutchan J. H. Jr, Lewis W. M. Jr, Kendall C. & McGrath C. C. Variation in trophic shift for stable isotope ratios of carbon, nitrogen, and sulfur. Oikos 102, 378–390 (2003).

[b64] Richards M. P., Fuller B. T., Sponheimer M., Robinson T. & Ayliffe L. Sulfur isotopes in palaeodietary studies: A review and results from a controlled feeding experiment. Intl. J. Osteoarchaeol. 13, 37–45 (2003).

[b65] Moreno R., Jover L., Munilla I., Velando A. & Sanpera C. A three-isotope approach to disentangling the diet of a generalist consumer: the yellow-legged gull in northwest Spain. Mar. Biol. 157, 545–553 (2010).

