# Supplementary material for: Major decline in marine and terrestrial animal consumption by brown bears (Ursus arctos)
Source: Sci Rep. 2015 Mar 17;5:9203. doi: 10.1038/srep09203 (PMC4361857; doi:10.1038/srep09203)
Supplement: Supplementary Information [file srep09203-s1.pdf]

Major decline in marine and terrestrial animal consumption by brown bears (*Ursus arctos*)

Jun Matsubayashi, Junko O. Morimoto, Ichiro Tayasu, Tsutomu Mano, Miyuki Nakajima, Osamu Takahashi, Kyoko Kobayashi, Futoshi Nakamura

Supplementary Information:

1. Supplementary Text

2. References for Supplementary Text

3. Supplementary Figures 1 to 2

4. Supplementary Tables 1 to 7

1. Supplementary Text

Correction of stable isotope values for the past diet resources of brown bears

We corrected for the expected historical differences in isotopic values in the diet items of brown bears. For the stable isotopes of carbon, a historical decrease in $\delta^{13}C$ due to the combustion of fossil fuels (Suess effect) is expected. Based on ice-core studies[1,2], atmospheric $\delta^{13}C$ has decreased 0.22‰ per decade since 1960 and 0.05‰ per decade between 1860 and 1960[3]. Therefore, we assumed that bears in Period 3 were killed in 2010 and bears in Period 2 were killed in 1930; we then increased the $\delta^{13}C$ of diet items by 1.60‰ for Period 1 and by 1.25‰ for Period 2 compared with the dietary values in

Period 3.

The $\delta^{15}N$ value is also expected to have changed historically due to anthropogenic atmospheric nitrogen (AAN) and biological nitrogen fixation (BNF; the conversion of N_2 gas to ammonium)[3]. For the marine prey, the maximum change in $\delta^{15}N$ resulting from the introduction of AAN is 0.03‰, while the maximum change in$\delta^{15}N$ resulting from BNF is 0.13‰. To prevent overestimation of the past trophic level of bears, we assumed a maximum decrease in $\delta^{15}N$ in the modern atmosphere, and we increased the $\delta^{15}N$ of salmon by 0.16‰ for Period 1 compared with the dietary values in Period 3. We corrected the historical differences in the isotopic values in the terrestrial prey items by comparing the isotope ratios between the ancient and modern bone collagen samples of Sika deer (*Cervus nippon*). The herbivorous Sika deer exclusively depends on C_3 plants, and therefore, the bone collagen of past Sika deer should record isotopic changes in the historical C_3 plants. Data for modern deer (AD1990 - AD2000) were obtained from Halley et al.[4], who analyzed stable isotope values in deer teeth collagen (N = 99). We converted the $\delta^{15}N$ values for deer teeth collagen to $\delta^{15}N$ values for bone collagen using following equation[4],

$$\delta^{15}N_{\text{bone collagen}} = \delta^{15}N_{\text{teeth collagen}} + 1.3‰$$

Data for ancient deer were obtained from several archaeological studies performed in

Hokkaido[5-9], and the data included isotopic data for past deer from BC4000 to AD1300 (N=15). The mean $\delta^{15}N$ values of the modern and archaeological deer samples were 4.4 ± 0.85 ‰ (mean ± SD) and 4.0 ± 1.3 ‰, respectively. The differences in the isotopic values between the modern and ancient terrestrial prey items of bears were calculated as follows:

$$\varDelta_{\text{modern-ancient}}\,\delta^{15}N_{\text{deer}} = 4.4‰ - 4.0‰ = 0.4‰,$$

Therefore, the $\delta^{15}N$ values of the C_3 plants, C_3 fruits and terrestrial animals were increased by 0.4‰ for Period 1 compared with the dietary values in Period 3.

There are no known reasons for a change in the $\delta^{34}S$ values from Periods 1 to 3; therefore, we did not correct the $\delta^{34}S$ values for each time period.

2. References for Supplementary Text

1. Francey, R. J., et al. A 1000-year high precision record of $\delta^{13}C$ in atmospheric CO_2. *Tellus B Chem. Phys. Meterol.* **51,** 170–193 (1999).
2. Chamberlain, C. P., et al. Pleistocene to recent dietary shifts in California condors. *Proc. Natl. Acad. Sci. USA* **102,** 16707–16711 (2005).
3. Wiley, A. E., et al. Millennial-scale isotope records from a wide-ranging predator show evidence of recent human impact to oceanic food webs. *Proc. Natl. Acad. Sci.*

USA **110,** 8972–8977 (2013).

4. Halley, D. J., Kaji, K. & Minagawa, M. Variation in stable isotope ratios of carbon and nitrogen in Hokkaido sika deer Cervus Nippon during 1990 - 2000: possible causes and implications for management. *Wildl. Biol.* **12,** 211-217(2006).
5. Naito, Y., et al. Quantitative evaluation of marine protein contribution in ancient diets based on nitrogen isotope ratios of individual amino acids in bone collagen: An investigation at the Kitakogane Jomon site. *Am. J. Phys. Anthropol.* **143,** 31–40 (2010).
6. Naito, Y., et al. Dietary reconstruction of the Okhotsk culture of Hokkaido, Japan, based on nitrogen composition of amino acids: implications for correction of ^{14}C marine reservoir effects on human bones. *Radiocarbon* **52,** 671–681 (2010).
7. Tsutaya T., et al. Isotopic evidence of dietary variability in subadults at the Usu-moshiri site of the Epi-Jomon culture, Japan. *J. Archaeol. Sci.* **40,** 3914–3925 (2013).
8. Tsutaya, et al. Carbon and nitrogen isotope analyses of human and dog diet in the Okhotsk culture: perspectives from the Moyoro site, Japan. *Anthropol. Sci.* **122,** 89–99 (2014).
9. Yoneda, M. & Gakuhari, T. Stable isotope analysis on the Jomon human and

mammal bones from the Tenneru 1 site, Kushiro, Hokkaido. in *Hokkaido Archaeological Research Center Research Reports,* No. **138,** (eds Hokkaido Archaeological Research Center) 404–407 (Hokkaido Archaeological Research Center, Sapporo, Japan, 2008) (In Japanese).

3. Supplementary Figures 1 to 2

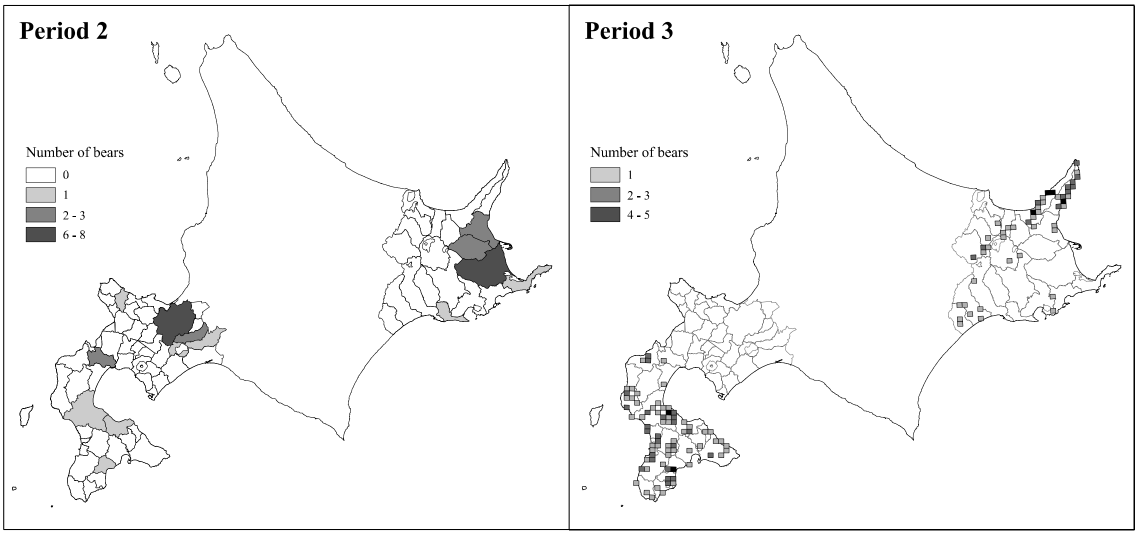

Supplementary Fig. 1. Capture locations of bears in Periods 2 and 3. Locations are shown at the municipality level for Period 2 and with a 5 km mesh for Period 3. This figure was made using GIS software (ArcGIS Desktop 10.2.1).

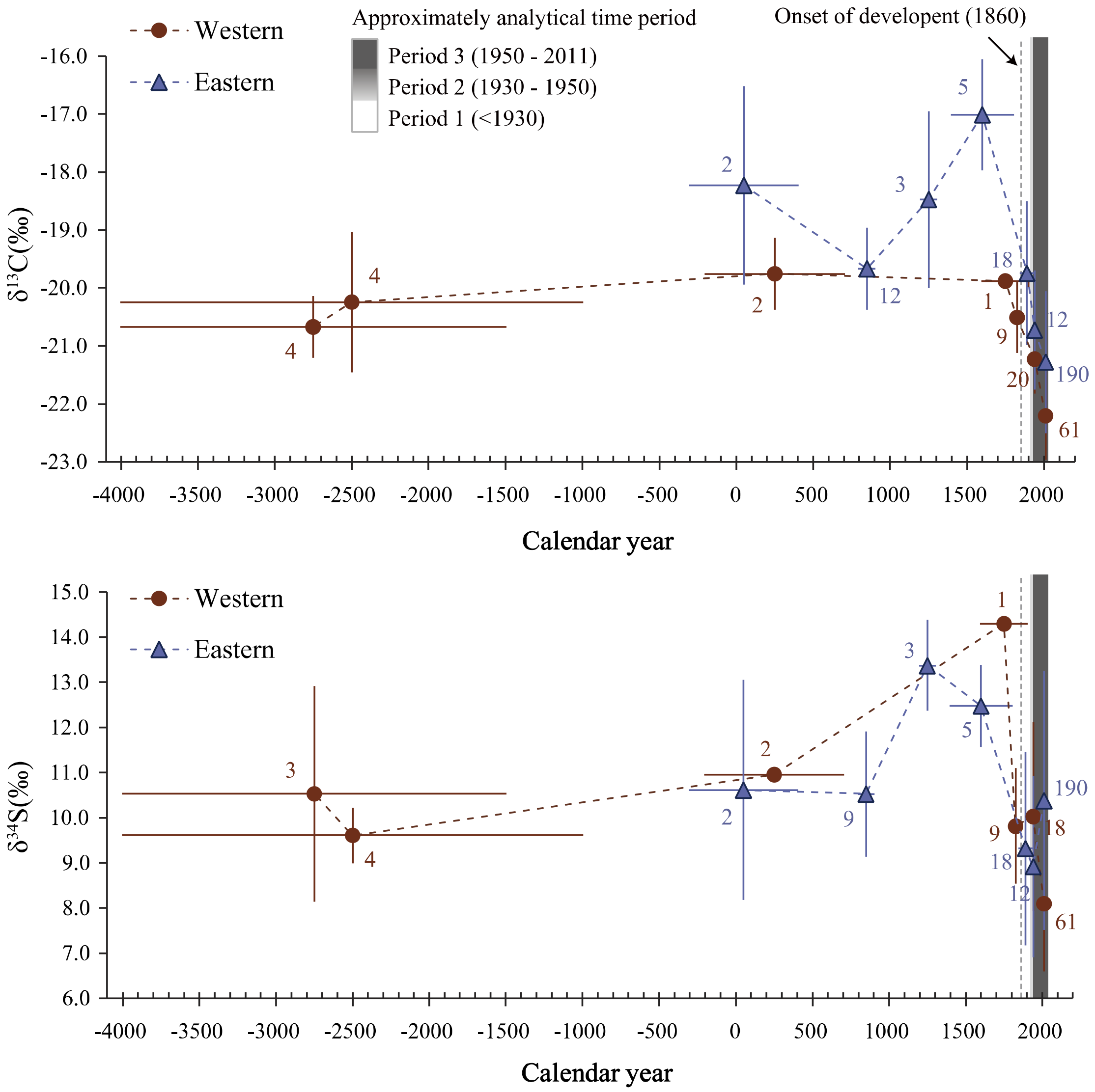

Supplementary Fig. 2. $\delta^{13}C$ and $\delta^{34}S$ values of historical bone collagen for two Hokkaido brown bear populations. The average age and isotopic composition of each time bin ± SD for the isotopic values are shown; the age range of each bear group is plotted with the sample size noted. The age of bear groups earlier than 1900 was determined according to the age of each archaeological site. The age of each site was determined using archaeological or geological information such as an excavated article or the geological age, respectively.

4. Supplementary Tables 1 to 7

Supplementary Table 1. Stable isotopes, C, N and S concentration data (mean ± SD), and sample size for bear bone collagen samples in each period.

Region	Time period	$\delta^{13}C$ (‰)	$\delta^{15}N$ (‰)	$\delta^{34}S$ (‰)	C (%)	N (%)	S (%)	N
Western	Period 1	-20.4 ± 0.8[a]	5.6 ± 1.5[a]	10.2 ± 1.7[a]	42.19 ± 2.60	15.03 ± 1.07	0.18 ± 0.02	20 (19)
	Period 2	-21.2 ± 0.6[b]	3.1 ± 1.2[b]	10.0 ± 2.1[a]	43.73 ± 1.89	15.63 ± 0.68	0.20 ± 0.03	20 (18)
	Period 3	-22.10 ± 1.2[c]	2.3 ± 1.1[b]	8.1 ± 1.5[b]	43.57 ± 1.63	15.81 ± 0.59	0.16 ± 0.02	61 (61)
Eastern	Period 1	-19.2 ± 1.5[a]	7.8 ± 2.9[a]	10.4 ± 2.2	43.24 ± 2.80	14.84 ± 1.16	0.19 ± 0.02	40 (37)
	Period 2	-20.7 ± 1.0[b]	5.2 ± 1.6[b]	8.9 ± 2.0	43.79 ± 0.56	15.68 ± 0.24	0.18 ± 0.02	12 (12)
	Period 3	-21.3 ± 1.2[b]	4.3 ± 2.4[b]	10.4 ± 2.9	43.26 ± 2.35	15.54 ± 0.83	0.16 ± 0.03	190 (190)

Numbers in parentheses are the sample sizes used for the sulfur stable isotope analysis.

Different letters indicate significance based on the Kruskal–Wallis test followed by the Steel–Dwass multiple comparisons test (α = 0.050).

Supplementary Table 2. Stable isotope values (mean ± SD) and sample size information for potential diet items of the Hokkaido brown bear.

		Western				Eastern			
Food resources	Parts	N	$\delta^{13}C$ (‰)	$\delta^{15}N$ (‰)	$\delta^{34}S$ (‰)	N	$\delta^{13}C$ (‰)	$\delta^{15}N$ (‰)	$\delta^{34}S$ (‰)
C_3 herbs									
Petasites japonicus	Stem	2	-29.4 ± 1.4	-1.4 ± 1.2	8.6 ± 1.7	2	-29.2 ± 0.2	-0.5 ± 0.9	6.7 ± 0.6
Angelica edulis	Stem and leaf	2	-29.5 ± 2.3	-2.0 ± 1.2	7.4 ± 2.1	0	-	-	-
Urtica platyphylla	Stem and leaf	2	-31.6 ± 3.2	-3.1 ± 2.1	6.2 ± 0.8	4	-28.6 ± 0.8	-5.8 ± 1.1	7.4 ± 0.2
Angelica ursina	Stem and leaf	2	-30.9 ± 2.6	-3.3 ± 0.4	8.6 ± 1.5	0	-	-	-
Angelica sachalinensis	Stem and leaf	2	-30.9 ± 1.8	-2.3 ± 0.2	7.1 ± 1.1	2	-29.6 ± 1.4	-4.0 ± 0.9	7.6 ± 1.0
Heracleum lanatum	Stem and leaf	2	-27.8 ± 0.5	-1.6 ± 0.2	6.6 ± 0.1	2	-28.7 ± 0.5	-4.2 ± 0.4	6.6 ± 0.1
Tilingia ajanensis	Stem and leaf	0	-	-	-	2	-29.8 ± 0.2	-4.2 ± 0.8	8.7 ± 0.7
C_3 fruits									
Fagus crenata	Carpology	2	-28.2 ± 1.3	-0.8 ± 2.1	9.5 ± 1.1	0	-	-	-
Quercus crispula	Carpology	2	-25.8 ± 0.5	-1.0± 1.0	8.2 ± 1.8	2	-25.8 ± 0.9	-1.0 ± 2.0	9.5 ± 1.9
Morus australis	Carpology	2	-28.7 ± 0.5	1.3 ± 0.2	12.6 ± 0.2	0	-	-	-
Vitis coignetiae	Carpology	1	-29.9	-3.0	4.8	2	-29.9 ± 0.5	-0.0 ± 1.1	7.7 ± 2.7
Aralia cordata	Carpology	1	-28.6	-1.0	7.7	3	-28.7 ± 0.9	-0.2 ± 0.6	7.8 ± 1.7
Sorbus commixta	Carpology	0	-	-	-	1	-28.9	-2.7	9.1
Corn									
Zea mays	Edible part	5	-11.0 ± 0.1	11.0 ± 1.1	1.8 ± 0.5	4	-11.2 ± 0.3	5.3 ± 1.1	2.4 ± 1.8
Terrestrial animals									
Lasius umbratus	Altogether	2	-26.6 ± 0.0	2.2 ± 0.1	5.3 ± 0.1	2	-28.4 ± 0.1	2.6 ± 0.0	3.9 ± 0.4
Formica yessensis	Altogether	2	-27.4 ± 0.7	3.1 ± 0.2	6.7 ± 2.4	2	-27.8 ± 0.0	4.7 ± 0.1	7.4 ± 0.6
Formica japonica	Altogether	2	-26.0 ± 0.1	2.8 ± 0.1	6.9 ± 0.1	0	-	-	-
Camponotus obscuripes	Altogether	2	-25.3 ± 02	1.2 ± 0.0	10.0 ± 0.1	0	-	-	-
Formica lemani	Altogether	2	-27.2 ± 0.4	4.1 ± 0.1	7.6 ± 0.3	0	-	-	-
Cervus nippon	Muscle	10	-26.8 ± 0.5	3.4 ± 1.0	8.9 ± 2.1	4	-26.5 ± 0.2	3.7 ± 0.9	2.4 ± 1.1
Salmon									
Oncorhynchus gorbuscha	Muscle	0	-	-	-	5	-21.2 ± 0.6	10.2 ± 0.4	18.4 ± 0.5
Oncorhynchus keta	Muscle	5		Common		5	-20.1 ± 0.6	11.7 ± 0.8	18.3 ± 0.4

"Common" indicates that the diet item shared isotopic data with anther region.

Supplementary Table 3. Steel-Dwass multiple comparisons test for isotopic values of Hokkaido brown bears among Period 1-3 in the eastern area (above diagonal) and western area (below diagonal).

		Period 1		Period 2		Period 3	
		t	*P*	*t*	*P*	*t*	*P*
$\delta^{13}C$	Period 1	—		3.149	0.005*	7.283	<0.001*
	Period 2	3.084	0.006*	—		1.668	0.218
	Period 3	6.183	<0.001*	4.902	<0.001*	—	
$\delta^{15}N$	Period 1	—		3.063	0.006*	6.561	<0.001*
	Period 2	4.166	<0.001*	—		1.739	0.191
	Period 3	6.199	<0.001*	2.152	0.080	—	
$\delta^{34}S$	Period 1	—		—		—	
	Period 2	0.175	0.983	—		—	
	Period 3	3.800	<0.001*	3.506	0.001*	—	

*Statistically significant difference ($\alpha = 0.050$)

Supplementary Table 4. Stable isotopes, and C, N and S concentration data (mean ± SD) for diet items of brown bears in each area.

Area	Items	N	$\delta^{13}C$ (‰)	$\delta^{15}N$ (‰)	$\delta^{34}S$ (‰)	C (%)	N (%)	S (%)
Western	C_3 plants	20	-29.2 ± 2.4	-1.6 ± 1.7	8.1 ± 2.2	41.20 ± 3.15	2.69 ± 2.05	0.15 ± 0.10
	Corn	5	-11.0 ± 0.1	10.6 ± 1.1	1.8 ± 0.5	45.51 ± 2.25	1.79 ± 0.12	0.06 ± 0.02
	Terrestrial animals	20	-26.7 ± 0.7	3.0 ± 1.0	8.1 ± 2.1	47.74 ± 3.39	11.93 ± 2.29	0.78 ± 0.03
	Salmon	5	-20.1 ± 0.6	11.7 ± 0.8	18.3 ± 0.4	49.00 ± 0.97	15.63 ± 0.32	0.98 ± 0.03
Eastern	C_3 herbs	12	-29.1 ± 0.9	-4.1 ± 2.0	7.7 ± 0.9	39.50 ± 4.27	4.82 ± 2.38	0.40 ± 0.12
	C_3 fruits	8	-28.3 ±1.7	0.0 ± 1.6	8.4 ± 2.1	42.40 ± 1.98	1.33 ± 0.32	0.08 ± 0.01
	Corn	4	-11.2 ± 0.3	5.3 ± 1.1	2.4 ± 1.8	42.61 ± 1.26	1.44 ± 0.10	0.06 ± 0.00
	Terrestrial animals	8	-27.3 ± 0.8	3.7 ± 1.0	5.0 ± 1.6	45.84 ± 1.46	11.51 ± 2.50	0.69 ± 0.24
	Salmon	10	-20.6 ± 0.8	11.0 ± 1.0	18.4 ± 0.5	47.74 ± 1.63	15.17 ± 0.56	0.96 ± 0.03

Supplementary Table 5. Contribution percentages of each diet item to the bears' diet estimated by SIAR.

Area	Time bin	C_3 plants	Corn	Terrestrial animals	Salmon
Western	Period 1	41.4 (19.6–61.5)[b]	NA	55.8 (34.7–77.7)[a]	3.2 (0.2–6.6)[a]
	Period 2	83.6 (69.1–92.7)[a]	6.3 (0.8–11.8)[a]	7.3 (0.0–24.5)[b]	1.0 (0.0–2.2)[a]
	Period 3	85.1 (82.2–87.9)[a]	9.6 (6.8–12.8)[a]	5.2 (2.3–7.9)[b]	0.0 (0.0–0.2)[a]

Area	Time bin	C_3 herbs	C_3 fruits	Corn	Terrestrial animals	Salmon
Eastern	Period 1	12.1 (0.0–27.2)[b]	1.0 (0.0–14.1)[a]	NA	64.1 (47.7–78.1)[a]	18.7 (14.8–22.5)[a]
	Period 2	35.2 (17.9–55.8)[ab]	29.6 (0.2–47.5)[a]	5.0 (0.6–8.9)[a]	26.5 (11.9–45.2)[b]	5.2 (1.7–9.7)[b]
	Period 3	53.9 (37.3–71.0)[a]	16.6 (0.0–41.2)[a]	9.3 (8.1–10.7)[a]	8.4 (3.3–13.0)[b]	8.2 (5.4–10.3)[b]

Median percentages, low and high 95% high-density ranges (hdr) are shown.

Different letters indicate significance based on the overlap of 95% hdr.

Supplementary Table 6. Information on archaeological sites where the ancient Hokkaido brown bear bone samples were excavated.

Region	Site	Site-ID	Age	N	Institute
Western	Setanai Chasi ato	STN	17C - 19C	1	Setana Town Historical Museum, Setana town
	Kotan Onsen	KO	BC4000 - BC1000	3	Yakumo Cultural Property Research Institute, Yakumo Board of Education
	Bifue Iwakage	B	Early-mid 19C	9	Chitose Cultural Property Research Institute, Chitose Board of Education
	Atsuma	ATM	BC4000 - BC1000	1	Chitose Cultural Property Research Institute, Chitose Board of Education
	Usu Moshiri	USM	BC2C - AD7C	2	Chitose Cultural Property Research Institute, Chitose Board of Education
	Irie Shell Midden	IRE	BC4000 - BC1500	4	Chitose Cultural Property Research Institute, Chitose Board of Education
Eastern	Sakaeura 2	TS	9C	12	Forest of the remains of place, Tokoro city
	Ikushina Kita Kaigan	IKS	15C - 18C	5	Archaeologist Center for Ainu & Indigenous Studies, Hokkaido University
	Otafuku Iwa	OTF	13C	3	Rausu Town Historical Museum, Rausu town
	Nusamai	KN	BC300 - AD400	2	Kushiro Cultural Property Research Institute, Kushiro Board of Education
	Nijibetsu-Suwan	NJB	Late 19C - early 20C	18	Department of Archaeology and Ethnology, Faculty of Letters, Keio University

Supplementary Table 7. Isotope values, C, N and S concentration, and museum specimen information for the past brown bear bone collagen.

Analysis No.	Site	Piece	Sex	Age	% collagen yield	C(%)	N(%)	S(%)	C/N	N/S	C/S	δ^{13}C(‰)	δ^{15}N(‰)	δ^{34}S(‰)	Time period
IKS1	Eastern	Skull	Male	Adult	6.5	43.79	14.90	0.22	3.43	154.71	454.59	-16.0	13.3	12.9	Period 1
IKS2	Eastern	Skull	Male	Adult	7.0	43.21	14.61	0.22	3.45	151.26	447.38	-16.0	13.5	14.0	Period 1
IKS3	*Eastern*	*Mandible*	*Male*	*Adult*	*3.3*	*43.39*	*13.67*	-	*3.70*	-	-	*-17.9*	*11.2*	-	*Period 1*
IKS4	Eastern	Skull	Male	Adult	4.6	44.17	14.64	0.24	3.52	141.04	425.42	-17.8	9.8	12.0	Period 1
IKS5	Eastern	Skull	Male	Adult	5.4	44.10	14.31	0.24	3.59	134.83	415.37	-18.4	10.6	11.7	Period 1
IKS6	*Eastern*	*Skull*	*Male*	*Adult*	*3.3*	*42.93*	*13.67*	-	*3.66*	-	-	*-17.8*	*12.0*	-	*Period 1*
IKS7	Eastern	Skull	Male	Adult	5.2	45.30	15.25	0.24	3.46	146.74	435.77	-16.9	12.0	11.7	Period 1
IKS8	*Eastern*	*Limb bones*	*Male*	*Adult*	*2.7*	*41.49*	*12.98*	-	*3.73*	-	-	*-19.0*	*10.2*	-	*Period 1*
OTF1	Eastern	Skull	Male	3	15.7	46.66	16.46	0.23	3.31	166.87	472.94	-18.4	5.6	12.2	Period 1
OTF2	Eastern	Skull	Male	4	12.2	45.83	16.12	0.21	3.32	171.65	487.94	-16.7	11.4	14.6	Period 1
OTF3	*Eastern*	*Skull*	*Male*	*8*	*2.1*	*41.80*	*13.08*	-	*3.73*	-	-	*-19.2*	*5.8*	-	*Period 1*
OTF4	*Eastern*	*Skull*	*Male*	*3*	*1.8*	*38.65*	*12.31*	-	*3.66*	-	-	*-20.5*	*4.7*	-	*Period 1*
OTF5	*Eastern*	*Skull*	*Female*	*3*	*2.2*	*38.61*	*12.33*	-	*3.65*	-	-	*-19.3*	*5.2*	-	*Period 1*
OTF6	*Eastern*	*Skull*	*Female*	*8*	*1.1*	*37.28*	*9.79*	-	*4.44*	-	-	*-20.2*	*7.8*	-	*Period 1*
OTF7	Eastern	Skull	Female	3	14.3	46.88	16.48	0.19	3.32	197.92	562.94	-20.4	2.6	13.3	Period 1
KN1	Eastern	Thighbone	-	-	15.3	45.68	16.03	0.23	3.33	157.28	448.27	-16.5	11.7	13.0	Period 1
KN8	Eastern	Thighbone	-	-	6.0	41.76	14.78	0.17	3.30	204.28	577.05	-19.9	5.8	8.2	Period 1
KN13	*Eastern*	*Thighbone*	-	-	*1.5*	*38.32*	*12.21*	-	*3.66*	-	-	*-17.6*	*14.9*	-	*Period 1*
TS30	Eastern	Mandible	-	Adult	1.7	39.36	13.01	-	3.53	-	-	-20.3	5.2	-	Period 1
TS31	Eastern	Mandible	-	Subadult	11.0	45.70	16.03	0.19	3.32	196.86	561.04	-19.5	7.8	12.2	Period 1
TS32	*Eastern*	*Mandible*	*Female*	*Adult*	*2.1*	*38.12*	*11.55*	-	*3.85*	-	-	*-20.6*	*5.5*	-	*Period 1*
TS33	Eastern	Mandible	-	Adult	0.9	36.64	12.09	-	3.54	-	-	-20.4	6.1	-	Period 1
TS35	Eastern	Mandible	-	Adult	4.2	42.03	13.83	0.20	3.55	155.49	472.76	-20.3	5.5	10.5	Period 1
TS36	*Eastern*	*Mandible*	-	*Adult*	*1.9*	*39.42*	*11.88*	-	*3.87*	-	-	*-20.4*	*7.7*	-	*Period 1*
TS37	Eastern	Mandible	Female	Adult	6.9	43.18	14.65	0.19	3.44	172.63	508.81	-20.2	7.5	11.9	Period 1
TS159	Eastern	Mandible	Female	Adult	3.3	43.63	14.85	0.20	3.43	168.12	493.93	-19.6	7.5	10.1	Period 1
TS163	*Eastern*	*Mandible*	*Female*	*Adult*	*1.7*	*40.39*	*12.35*	-	*3.81*	-	-	*-20.4*	*6.8*	-	*Period 1*
TS167	Eastern	Mandible	Male	Adult	3.1	41.28	13.96	0.18	3.45	172.54	510.29	-19.4	6.8	7.7	Period 1
TS175	*Eastern*	*Mandible*	*Male*	*Adult*	*2.4*	*42.13*	*13.59*	-	*3.62*	-	-	*-18.7*	*10.5*	-	*Period 1*
TS181	*Eastern*	*Mandible*	-	*Adult*	*3.2*	*41.38*	*12.89*	-	*3.75*	-	-	*-20.1*	*6.4*	-	*Period 1*
TS189	Eastern	Mandible	-	Adult	2.7	40.41	13.36	-	3.53	-	-	-19.9	8.2	-	Period 1
TS218	Eastern	Mandible	-	-	4.1	43.74	14.75	0.17	3.46	195.96	581.04	-18.7	10.3	11.1	Period 1
TS235	Eastern	Mandible	Female	Adult	3.6	42.67	14.32	0.21	3.48	155.87	464.51	-18.0	11.7	11.4	Period 1
TS236	Eastern	Mandible	-	Adult	3.6	41.66	14.35	0.20	3.39	162.05	470.27	-19.8	5.6	8.8	Period 1
TS269	*Eastern*	*Mandible*	*Male*	*Adult*	*1.4*	*36.53*	*10.16*	-	*4.20*	-	-	*-19.5*	*11.1*	-	*Period 1*
TS356	*Eastern*	*Mandible*	-	-	*0.7*	*32.04*	*5.18*	-	*7.21*	-	-	*-23.5*	*9.0*	-	*Period 1*
TS358	Eastern	Mandible	-	-	3.7	41.66	13.96	0.20	3.48	155.66	464.58	-19.9	6.9	11.1	Period 1
TS359	*Eastern*	*Mandible*	*Female*	*Adult*	*2.9*	*40.15*	*12.51*	-	*3.74*	-	-	*-20.6*	*5.1*	-	*Period 1*
NJB4	Eastern	Mandible	Female	Adult	7.3	42.57	14.14	0.23	3.51	139.18	418.94	-21.7	4.6	8.2	Period 1
NJB9	Eastern	Mandible	Male	Adult	7.0	42.78	14.72	0.23	3.39	143.48	417.07	-18.7	8.9	9.0	Period 1
NJB17	Eastern	Mandible	Male	Adult	7.7	41.78	14.41	0.23	3.38	142.38	412.70	-17.9	10.3	11.4	Period 1
NJB18	Eastern	Mandible	Female	Adult	5.7	31.94	11.06	0.23	3.37	111.91	323.36	-21.0	5.7	9.2	Period 1
NJB42	Eastern	Mandible	Female	Adult	11.0	45.60	16.17	0.22	3.29	164.78	464.82	-19.0	7.2	10.8	Period 1
NJB45	Eastern	Mandible	Female	Adult	9.3	45.26	15.81	0.22	3.34	163.66	468.38	-20.6	6.6	11.3	Period 1
NJB60	Eastern	Mandible	Male	Adult	8.6	43.61	15.34	0.22	3.32	157.10	446.75	-17.8	10.2	12.7	Period 1
NJB61	Eastern	Mandible	Female	Adult	4.7	42.50	14.33	0.26	3.46	127.37	377.89	-20.1	5.7	9.2	Period 1
NJB84	Eastern	Mandible	Male	Adult	4.7	44.15	14.78	0.25	3.48	136.82	408.60	-18.4	10.8	10.6	Period 1
NJB87	Eastern	Mandible	Male	Adult	7.8	46.78	16.61	0.22	3.28	169.87	478.30	-19.4	7.6	10.0	Period 1
NJB88	Eastern	Mandible	Female	Adult	9.9	44.89	15.81	0.22	3.31	164.47	466.81	-20.6	5.4	7.0	Period 1
NJB89	Eastern	Mandible	Male	Adult	7.9	45.68	16.41	0.21	3.25	177.86	495.07	-20.4	5.2	5.3	Period 1
NJB99	Eastern	Mandible	Male	Adult	7.0	45.57	15.21	0.22	3.50	156.35	468.50	-19.4	8.7	9.5	Period 1
NJB111	Eastern	Mandible	Female	Adult	5.4	43.23	15.17	0.23	3.32	152.27	433.93	-19.7	5.7	9.0	Period 1
NJB117	Eastern	Mandible	Female	Adult	12.9	46.02	16.21	0.23	3.31	161.92	459.57	-22.0	1.1	7.5	Period 1
NJB118	Eastern	Mandible	Male	Adult	7.8	44.15	15.43	0.25	3.34	139.69	399.76	-21.2	4.2	4.6	Period 1
NJB146	Eastern	Mandible	Female	Adult	7.7	43.49	14.88	0.22	3.41	151.39	442.53	-18.9	9.6	11.8	Period 1
NJB2R	Eastern	Mandible	Female	Adult	4.1	40.41	14.25	0.24	3.31	137.03	388.62	-18.7	8.9	10.7	Period 1
KO1	Western	Thighbone	-	-	6.1	45.01	15.86	0.20	3.31	184.17	522.71	-18.6	9.7	10.5	Period 1
KO2	Western	Thighbone	-	-	5.7	44.19	15.64	0.18	3.30	197.78	558.88	-21.2	4.3	8.8	Period 1
KO3	Western	Thighbone	-	-	4.2	35.11	12.35	0.18	3.32	157.35	447.21	-21.5	3.0	9.6	Period 1
STN	Western	Scapula	-	-	7.0	41.85	14.53	0.20	3.36	168.30	484.88	-19.9	5.8	14.3	Period 1
ATM	Western	Mandible	-	-	6.4	42.44	14.91	0.18	3.32	190.06	540.82	-19.6	4.7	9.6	Period 1
USM2	Western	Thighbone	-	-	4.9	42.61	15.16	0.18	3.28	193.97	545.32	-19.2	6.0	11.0	Period 1
USM3	Western	Patella	-	-	9.1	43.68	15.24	0.21	3.34	163.97	470.01	-20.4	3.9	10.9	Period 1
IRE23	Western	Humerus	-	-	9.7	42.51	15.44	0.22	3.21	161.96	446.01	-21.1	3.5	11.2	Period 1
IRE25	Western	Humerus	-	-	1.2	35.15	11.85	-	3.46	-	-	-21.3	4.4	-	Period 1
IRE26	Western	Radius	-	-	6.7	41.59	14.85	0.17	3.27	200.77	562.27	-20.2	4.8	7.3	Period 1
IRE27	Western	Radius	-	-	5.4	39.91	14.31	0.14	3.25	232.01	646.86	-20.1	5.3	13.0	Period 1
B1	Western	Skull	Male	22	7.8	42.78	15.33	0.17	3.26	207.41	578.90	-20.8	5.5	8.0	Period 1
B2	Western	Skull	Male	12	6.8	43.92	15.73	0.17	3.26	217.92	608.27	-21.1	6.2	7.8	Period 1
B3	Western	Skull	Male	22	7.3	42.95	15.58	0.20	3.22	182.44	502.83	-19.5	7.5	10.1	Period 1
B5	Western	Skull	Female	12	8.4	44.22	16.00	0.18	3.22	202.40	559.25	-20.6	4.2	11.3	Period 1
B7	Western	Skull	Female	16	16.1	42.56	15.39	0.17	3.23	201.79	558.22	-19.9	6.5	10.0	Period 1
B8	Western	Skull	Female	10	16.0	43.23	15.31	0.15	3.29	226.69	640.10	-21.3	4.2	10.8	Period 1

B9	Western	Skull	Female	14	14.7	43.39	15.59	0.15	3.25	230.24	640.62	-19.8	5.4	11.3	Period 1
B10	Western	Skull	Female	12	10.8	42.32	15.39	0.17	3.21	205.35	564.60	-20.7	3.8	8.9	Period 1
B14	Western	Skull	-	Adult	15.4	44.39	16.13	0.19	3.21	197.75	544.34	-20.8	4.2	10.1	Period 1
47708	*Eastern*	*Skull*	-	*Adult*	*0.9*	*35.98*	*10.86*	-	*3.87*	-	-	*-21.3*	*6.4*	-	*Period 2*
48853	Eastern	Skull	-	Adult	19.3	44.12	16.06	0.18	3.21	200.52	550.98	-21.4	2.8	6.4	Period 2
48870	Eastern	Skull	Female	Adult	19.6	43.27	15.72	0.18	3.21	196.51	540.98	-18.7	8.2	10.9	Period 2
48871	Eastern	Skull	-	7	23.1	44.70	15.82	0.18	3.30	195.47	552.33	-22.3	5.4	9.0	Period 2
48872	Eastern	Skull	Male	Adult	21.1	44.15	15.34	0.15	3.36	228.52	657.84	-22.0	6.4	10.7	Period 2
48873	Eastern	Skull	Male	Adult	21.2	42.50	15.36	0.19	3.23	188.50	521.63	-20.8	5.2	9.6	Period 2
48874	Eastern	Skull	-	Adult	22.8	44.18	16.10	0.19	3.20	192.04	527.07	-21.7	3.2	6.7	Period 2
48875	Eastern	Skull	-	Adult	22.5	43.72	15.76	0.19	3.24	185.53	514.57	-20.6	5.3	6.6	Period 2
48876	Eastern	Skull	Male	Adult	11.7	44.29	15.47	0.15	3.34	243.21	696.41	-20.2	5.1	10.9	Period 2
48877	Eastern	Skull	-	Adult	22.5	43.84	15.72	0.19	3.25	191.54	534.05	-21.2	2.3	7.8	Period 2
48878	Eastern	Skull	-	Adult	19.3	43.19	15.40	0.18	3.27	190.29	533.65	-19.7	6.5	11.6	Period 2
48879	Eastern	Skull	-	Adult	24.0	43.71	15.69	0.20	3.25	182.56	508.58	-20.6	5.3	6.1	Period 2
48880	Eastern	Skull	-	Adult	20.4	43.75	15.75	0.20	3.24	182.70	507.36	-19.6	6.3	10.7	Period 2
13112	Western	Skull	-	Adult	22.6	43.56	15.34	0.19	3.31	183.56	521.13	-20.9	4.2	7.8	Period 2
43096	Western	Skull	Female	Adult	19.4	43.08	15.64	0.20	3.21	182.68	503.25	-20.9	3.6	13.4	Period 2
47701	Western	Skull	-	Adult	20.7	43.30	15.70	0.18	3.22	194.04	535.11	-20.7	6.1	8.8	Period 2
47703	Western	Skull	-	Adult	20.8	43.28	15.55	0.15	3.25	241.96	673.46	-21.2	2.6	7.8	Period 2
48794	Western	Skull	-	Adult	16.7	43.45	15.82	0.20	3.20	180.08	494.54	-20.8	3.0	10.3	Period 2
48795	Western	Skull	Male	Adult	22.7	43.20	15.70	0.24	3.21	149.89	412.53	-21.4	2.3	10.4	Period 2
48796	Western	Skull	-	Adult	18.0	42.60	15.20	0.25	3.27	138.93	389.38	-20.8	3.4	11.4	Period 2
48797	Western	Skull	-	Adult	19.2	51.29	18.30	0.23	3.27	182.57	511.68	-20.7	2.8	14.3	Period 2
48801	Western	Skull	Male	Adult	19.0	43.01	15.32	0.23	3.27	152.61	428.29	-21.4	3.4	13.1	Period 2
48803	Western	Skull	Female	Adult	20.3	43.78	15.91	0.23	3.21	155.40	427.61	-20.7	3.6	11.6	Period 2
48805	Western	Skull	-	Adult	10.8	43.07	15.62	0.19	3.22	192.28	530.26	-20.9	2.4	8.1	Period 2
48807	Western	Skull	Male	Adult	6.3	41.79	14.57	0.19	3.35	177.56	509.18	-21.8	2.3	7.1	Period 2
48808	Western	Skull	-	Adult	17.5	42.19	15.24	0.19	3.23	187.33	518.56	-21.7	1.8	9.2	Period 2
48811	Western	Skull	-	Adult	20.3	43.75	15.75	0.19	3.24	187.94	522.05	-21.0	2.5	8.4	Period 2
48813	Western	Skull	-	Adult	19.8	42.49	15.29	0.19	3.24	185.50	515.35	-21.7	2.2	8.4	Period 2
48814	Western	Skull	Male	Adult	20.1	44.53	15.71	0.19	3.31	192.06	544.30	-22.1	1.4	9.2	Period 2
48816	Western	Skull	-	Adult	19.8	42.77	15.51	0.19	3.22	182.89	504.21	-20.3	5.6	9.5	Period 2
48818	Western	Skull	Female	Adult	17.4	44.37	15.55	-	3.33	-	-	-20.9	2.6	-	Period 2
48819	Western	Skull	-	Adult	19.5	44.81	15.28	-	3.42	-	-	-22.5	2.4	-	Period 2
48820	Western	Skull	-	Adult	20.8	44.33	15.59	0.15	3.32	241.46	686.69	-22.3	3.8	11.5	Period 2

The italicised samples showed beyond acceptable C/N ratios (2.9 – 3.6) and were not used for any other analysis.
